# A multi-centre randomized controlled trial investigating Consolidation Chemotherapy with and without oxaliplatin in distal rectal cancer and Watch & Wait

**DOI:** 10.1186/s12885-023-10984-2

**Published:** 2023-06-14

**Authors:** Angelita Habr-Gama, Guilherme Pagin São Julião, Cinthia D. Ortega, Bruna Borba Vailati, Sergio Araujo, Thiago Jorge, Jorge Sabbaga, Gustavo L. Rossi, Renata D’Alpino, Fabio Roberto Kater, Patricia Bailão Aguilar, Adrian Mattacheo, Rodrigo Oliva Perez, Latin American Rectal Cancer Consortium (LARCC)

**Affiliations:** 1 University of São Paulo School of Medicine, São Paulo, Brazil; 2 Angelita and Joaquim Gama Institute, Praça Amadeu Amaral, 47 – conj.111, São Paulo, 01327-904 Brazil; 3 Department of Coloproctology, Hospital Alemão Oswaldo Cruz, Praça Amadeu Amaral, 47 – conj.111, São Paulo, 01327-904 Brazil; 4 Department of Surgical Oncology, Hospital Beneficencia Portuguesa, Praça Amadeu Amaral, 47 – conj.111, São Paulo, 01327-904 Brazil; 5 Department of Radiology, Hospital das Clinicas HCFMUSP, Faculdade de Medicina, Universidade de Sao Paulo, São Paulo, Brazil; 6 Department of Radiology and Diagnostic Imaging, Hospital Israelita Albert Einstein, São Paulo, Brazil; 7 Department of Medical Oncology, Hospital Alemão Oswaldo Cruz, São Paulo, Brazil; 8 Department of Medical Oncology, Hospital Sírio Libanês, São Paulo, Brazil; 9 Servicio Cirugia General, Hospital Italiano de Buenos Aires, Sector de Coloproctologia, Buenos Aires, Argentina; 10 Centro Paulista de Oncologia, São Paulo, Brazil; 11 Department of Medical Oncology, Hospital Beneficencia Portuguesa, São Paulo, Brazil; 12 Department of Radiation Oncology, Hospital Alemão Oswaldo Cruz, São Paulo, Brazil; 13 Hospital Gral. J. M. Ramos Mejia, Buenos Aires, Argentina; 14 Ludwig Institute for Cancer Research, Praça Amadeu Amaral, 47 – conj.111, São Paulo, 01327-904 Brazil

**Keywords:** Rectal Cancer, Complete Clinical Response, Watch and Wait, Total neoadjuvant treatment, Consolidation chemotherapy

## Abstract

**Background:**

Neoadjuvant chemoradiation(nCRT) has been considered the preferred initial treatment strategy for distal rectal cancer. Advantages of this approach include improved local control after radical surgery but also the opportunity for organ preserving strategies (Watch and Wait-WW). Consolidation chemotherapy(cCT) regimens using fluoropyrimidine-based with or without oxalipatin following nCRT have demonstrated to increase complete response and organ preservation rates among these patients. However, the benefit of adding oxaliplatin to cCT compared to fluoropirimidine alone regimens in terms of primary tumor response remains unclear. Since oxalipatin-treatment may be associated with considerable toxicity, it becomes imperative to understand the benefit of its incorporation into standard cCT regimens in terms of primary tumor response. The aim of the present trial is to compare the outcomes of 2 different cCT regimens following nCRT (fluoropyrimidine-alone versus fluoropyrimidine + oxaliplatin) for patients with distal rectal cancer.

**Methods:**

In this multi-centre study, patients with magnetic resonance-defined distal rectal tumors will be randomized on a 1:1 ratio to receive long-course chemoradiation (54 Gy) followed by cCT with fluoropyrimidine alone versus fluoropyrimidine + oxaliplatin. Magnetic resonance(MR) will be analyzed centrally prior to patient inclusion and randomization. mrT2-3N0-1 tumor located no more than 1 cm above the anorectal ring determined by sagittal views on MR will be eligible for the study. Tumor response will be assessed after 12 weeks from radiotherapy(RT) completion. Patients with clinical complete response (clinical, endoscopic and radiological) may be enrolled in an organ-preservation program(WW). The primary endpoint of this trial is decision to organ-preservation surveillance (WW) at 18 weeks from RT completion. Secondary endpoints are 3-year surgery-free survival, TME-free survival, distant metastases-free survival, local regrowth-free survival and colostomy-free survival.

**Discussion:**

Long-course nCRT with cCT is associated with improved complete response rates and may be a very attractive alternative to increase the chances for organ-preservation strategies. Fluoropyrimidine-based cCT with or without oxaliplatin has never been investigated in the setting of a randomized trial to compare clinical response rates and the possibility of organ-preservation. The outcomes of this study may significantly impact clinical practice of patients with distal rectal cancer interested in organ-preservation.

**Trial registration:**

www.clinicaltrials.gov NCT05000697; registered on August 11^th^, 2021.

## Background

Significant tumor response to neoadjuvant chemoradiation (CRT) therapy has resulted in a dramatic change in distal rectal cancer management [1]. Observation of near-complete and complete tumor response to treatment has led surgeons to consider organ-preservation strategies to avoid the need for radical surgery [2–5]. Patients that achieve clinical complete response (cCR) defined by clinical, endoscopic and radiological criteria have been managed non-operatively and enrolled in a strict surveillance program (Watch and Wait – WW) with acceptable oncological outcomes [6–9]. These patients would ultimately avoid the risk of immediate postoperative morbidity and mortality in addition to the potential negative consequences of urinary, sexual and anorectal function frequently seen after total mesorectal excision (TME) [10–12]. In addition, patients managed by WW would avoid the need for a temporary/definitive stoma – particularly relevant among patients with distal tumors [13]. In this latter group of patients, abdominal perineal resections (APR) with a definitive stoma or intersphincteric resections (ISR) with poor postoperative function are the surgical alternatives [14, 15].

In this setting, several attempts have been made to increase chances for achieving a clinical complete response and allowing for the opportunity of entering an organ-preserving pathway [16]. Several treatment-related features may affect complete tumor regression rates to neoadjuvant strategies in rectal cancer [17–19]. There is data to suggest that specific characteristics of both radiation and chemotherapy may influence complete tumor regression rates. Data provided from multiple studies using different radiation doses in rectal cancer suggests that there is an increase in complete response rates as total doses increase [18]. Radiosensitizing chemotherapy may also affect response rates to neoadjuvant treatment strategies. Incorporation of additional chemotherapy following radiation completion (consolidation chemotherapy) has also shown to significantly increase complete clinical and pathological response rates [16, 19–21]. However, none of these studies were specifically designed to compare different consolidation chemotherapy regimens. Regimens including exclusively 5FU-based or 5FU + oxaliplatin regimens have both been used in different studies using consolidation chemotherapy after nCRT completion suggesting higher rates of pathological complete response (pCR) or cCR compared to historical cohorts [19]. However, there has never been a head-to-head comparison between 5FU-based alone versus 5FU-oxaliplatin in consolidation regimens. Earlier studies attempting to incorporate concomitant oxaliplatin into standard nCRT regimens failed to demonstrate benefits in pCR rates while did result in excessive toxicity associated with the use of oxaliplatin [22]. However, when offered in a consolidation regimen, oxalipatin could potentially decrease its associated toxicity while effectively providing significant increase in response rates. For these reasons, we aimed to compare the outcomes of fluoropyrimidine-only consolidation chemotherapy to fluoropyrimidine + oxaliplatin in achieving a cCR after nCRT in this prospective randomized clinical trial.

## Patients and methods

Patients with distal rectal cancer will be eligible for the study after initial clinical, endoscopic and radiological assessment. At this point, patients will be offered to participate in the study and after informed consent, randomized to study's arms as follows.

## Eligibility and inclusion criteria

Patients will be eligible in the presence of the following inclusion criteria:

1. Age ≥18 years;
2. ECOG 0-2 or KPS≥70;
3. Primary rectal adenocarcinoma (biopsy confirmed) within the reach of digital rectal examination (at least lower tip/border) by the attending colorectal surgeon;
4. Endoscopic documentation of the primary tumor;
5. Abdominal and chest computed tomography (CT) scans showing no evidence of metastatic disease;
6. High-resolution magnetic resonance images performed at either 1.5T or 3.0T system using a phased array surface coil with: sagittal T2 images including the anal verge and the sacrum; axial oblique T2 weighted images acquired in a plane perpendicular to the long axis of the rectal wall guided by the sagittal images; coronal images acquired in parallel to the anal canal plane. Small field of view (16-18cm), 3mm section thickness, increased matrix size and increased number of signal averages are required;
7. Radiological defining criteria (centralized):
  - Lower edge of tumor at the level (max. 1cm distance) or below the anorectal ring defined at sagittal or coronal views;
  - mrT2, mrT3 (any subclassification)
  - mrN0-1 (≤3 radiologically positive lymph nodes)
  - mr Extramural Vascular Invasion (EMVI): any status
  - mr Mesorectal Fascia (MRF): any status
8. Tumor location (both criteria need to be met in all patients)*:
  - Lower edge of tumor at the level (max. 1cm distance) or below the anorectal ring defined at sagittal or coronal views using magnetic resonance;
  - Primary rectal adenocarcinoma (biopsy confirmed) within the reach of digital rectal examination (at least lower tip/border) by the attending colorectal surgeon

* Tumor height will be defined by an objective and reproducible criteria (distance of the lower edge of the tumor from the insertion of the levator ani muscles – anorectal ring – determined on sagittal and/or coronal views from MR T2-weighted sequences). As MR images will be centrally reviewed and stored, this will provide an objective, reproducible and allow auditing of the data. Accessibility to the finger of the examining physician will NOT be used as a measure of height—instead, only used to provide homogeneity in tumor response assessment criteria for all patients included in the study.

### Exclusion criteria

1. Pregnancy
2. ECOG ≥ 3 or KPS < 70
3. Unwilling to consent
4. Metastatic disease (any kind; internal iliac and obturator nodes are considered local disease and not metastatic disease and therefore will not be considered as exclusion criteria)
5. mrT4 or mrN2
6. Previous pelvic irradiation
7. Baseline neuropathy
8. Receiving treatment of other anti-cancer drug or methods
9. Presence of uncontrolled life threatening diseases

## Endpoints

Analysis of the endpoints will be performed on intention-to-treat.

### Primary endpoint

Decision to Watch and Wait due to clinical complete response achieved at 18 weeks from last date of radiation using clinical (Digital rectal examination—DRE), endoscopic and radiological criteria (mrTumor Regresion Grade—TRG grade) or near-complete clinical response (no progressive disease clinically, endoscopically or radiologically).

Definition of cCR available below and at the discretion of the attending surgeon.

Definition of radiological complete response as described below (centralized).

Patients will be counted as event if at 18 weeks the decision is to interrupt Watch and Wait and proceed to surgery (any kind) because of overt incomplete clinical response. In order to standardize assessment of response and reduce inter-observer variability, the decision to continue on Watch and Wait (or not) will be at the discretion of the central committee during central revision of studies.

### Secondary endpoints

- Surgery-free survival at 3 years
- TME-free survival at 3 years
- Distant metastases free survival at 3 years
- Local regrowth-free survival at 3 years
- Colostomy-free survival at 3 years

## Definitions

### Definition of cCR

- Endoscopic: white scar, teleangiectasia, absence of ulceration and/or mass [6].
- Clinical: no irregularity, firm area with minor induration [6].
- Radiological: mrTRG1: fibrosis with low signal intensity seen on T2 weighted images replacing the primary tumor; no restricted diffusion on diffusion weighted images; no nodes with border irregularity or mixed signal intensity; no EMVI [23–26].

### Definition of near-complete response

- Endoscopic: residual tumor size ≤ 2 cm (or reduction of ≥ 70% original tumor volume/size) [4, 27, 28].
- Clinical: only superficial ulceration or minor (questionable) irregularities of the mucosal/rectal wall.
- Radiological: mrTRG2 predominant fibrosis with low signal with foci of intermediate tumor signal intensity seen on T2 weighted images with or without restricted diffusion; mrTRG1: fibrosis with low signal intensity seen on T2 weighted images replacing the primary tumor with restricted diffusion; no nodes with border irregularity or mixed signal intensity; no EMVI [27].

### Central Committee

The central committee is a multidisciplinary group of surgeons, medical oncologists and radiologists previously appointed at the beginning of the recruitment of patients and with previous experience with organ preservation [2, 21]. This group of specialists will be responsible to assess the baseline staging and define if the patients fulfill all the inclusion/exclusion criteria prior to randomization. The committee will also be responsible to evaluate the endoscopic and radiological tumor re-assessment studies at 12 and 18 weeks from radiation completion. The definition of the primary endpoint to suggest continuation on the Watch and Wait pathway or not will be at the discretion of this committee. The final decision to Watch and Wait or any alternative surgical decision will be entirely at the discretion of each institution.

## Technical aspects of assessment tests

### Suggested MR protocol

1.5 T—FRFSE; TR/TE: 3300/120 (ms); slice thickness/gap: 3.0/0; Matrix: 256 × 256; NSA 8

3.0 T – FRFSE; TR/TE: 8000/150 (ms); slice thickness/gap: 3.0/0; Matrix: 288 × 288; NSA 5

DWI – inclusion of a high b-value of at least 800

### Suggested endoscopic assessment

Endoscopic assessment using a flexible scope (gastroscope preferred for retroflexion); direct endoscopic and retroflexion view of the primary tumor/scar; endoscopic biopsies at discretion of participating center.

## Treatment arms

1. RT (54 Gy) plus daily concomitant capecitabine 825 mg/m2 bid, followed by mFOLFOX6 or XELOX for 4 cycles (12 weeks), starting 1 week after radiotherapy ended;
2. RT (54 Gy) plus daily concomitant capecitabine 825 mg/m2 bid, followed by capecitabine 2000 mg/m2/day for 14 days in a 21 days cycle for 4 cycles (12 weeks), starting 1 week after radiotherapy ended;

## Radiotherapy

Radiotherapy will be delivered on a linear accelerator in prone or supine position, preferably with full bladder. The use of a belly board is allowed. Isocentric 3 or 4 fields, as well as an IMRT technique is allowed, as long as all beams are treated on a daily basis. The dose distribution and calculation should be performed on CT or MR and specified according to the ICRU 50 guidelines.

### Dose specification

All patients will receive 25 daily fractions of 1.8 Gy up to a total dose of 45 Gy to the pelvic field including the tumor bed with a margin and the regional lymph nodes. A field reduction after 45 Gy is recommended up to 54 Gy. The last 5 fractions will then be given to the tumor bed with a margin.

### Target volume

#### Pelvic CTV

- The primary tumor
- Mesorectum: Distally, only lymph nodes or tumor deposits up to 4 cm are included. For tumors in lower rectum this means that the entire mesorectum down to the pelvic floor is included.
- Presacral nodes and nodes along the rectal superior artery: Since local recurrences are very unusual above S1 – S2, lymph nodes above this level should not be included unless there are signs of radiologically positive lymph nodes presacrally. If this is the case, the cranial limit of CTV should be at least 1 cm above the most cranial radiologically positive lymph node.
- Lateral lymph node stations: Until they reach the level of the obturator canal Internal iliac artery up to the bifurcation from the external iliac artery. The cranial border for the CTV is in most cases just below the bifurcation of the internal and external iliac arteries. In most patients this is at the level of S1 – S2.
- Ischio-rectal fossa and the anal canal: Included in pelvic CTV only if the tumor grows into the levators or down into the anal canal.

#### Boost GTV

GTV is the visible primary tumor and radiologically positive lymph nodes.

#### CTV boost

GTV boost plus a margin of 2 cm within the same anatomical compartment as the tumour is in, for the dose of 45 Gy, also around radiologically enlarged lymph nodes.

#### PTV

The above description relates to the CTV. A PTV should normally be defined and includes CTV and internal target volume (ITV) and a margin necessary for the setup. These margins are depending upon several factors that are related to the equipment at each radiotherapy center.

## Chemotherapy protocols

### Concomitant chemotherapy

Concomitant capecitabine: 825 mg/m2 bid on the radiotherapy days only.

### Consolidation chemotherapy

1. Consolidation capecitabine (alone): 1000 mg/m2 bid, for 14 days, in a 3 week cycle, for 4 cycles
2. Consolidation options with oxaliplatin: 
  2.1. mFOLFOX6: Oxaliplatin 85 mg/m2 plus Leucovorin 400 mg/m2 on a concomitant 2 h infusion. 5FU 400 mg/m2 on a bolus infusion, followed by 5FU 2400 mg/m2 in 46 h infusion, every 2 weeks, for 6 cycles
  2.2. CAPOX: Oxaliplatin 130 mg/m2 on a 2 h infusion. Capecitabine 1000 mg/m2 bid daily, for 14 days, starting on the evening of the oxaliplatin infusion. Repeat every 3 weeks, for 4 cycles

## Toxicities

### Chemotherapy toxicity and dose adjustment

Dose reduction is planned in case of severe haematological and/or non haematological toxicities. Dose adjustments are to be made according to the system showing the greatest degree of toxicity. Toxicities will be graded using the NCI CTC, version 5.0. Treatment will be delayed until: neutrophils ≥ 1.5 × 10^9^ /L and platelets ≥ 75 × 10^9^ /L.

1. 5FU dose-modification:
  Recovery from mucositis, diarrhea. If 5-FU treatment is delayed, then the associated oxaliplatin dose should also be delayed. If 5-FU is discontinued permanently, oxaliplatin should also be discontinued. If toxicity requires a dosing delay of more than four weeks, the subject will be permanently withdrawn from the study treatment for toxicity. Dose modifications for hematologic or gastrointestinal (GI) toxicity will be based on the worst toxicity observed during the previous cycle. After recovery, standard dose adjustments for 5-FU toxicity should be applied. The dose of 5-FU should be reduced by 20% in subsequent cycles for the following toxicities: febrile neutropenia, grade 4 thrombocytopenia, or failure of hematological recovery to neutrophils ≥1500 /mL and platelets ≥75000/mL within 2 weeks of the scheduled start of the next treatment cycle; grade 3-4 mucositis, diarrhea, or nausea or vomiting in spite of optimal antiemetic prophylaxis. In the mFOLFOX6 protocol, the bolus 5FU should be interrupted before the aforementioned reduction of the continuos infusion 5FU. A second dose reduction of 5-FU of 20% from the original dose may be made if the above toxicities recur. After reductions of doses, they should not be increased again and must be carried on to the rest of the treatment.
2. Capecitabine:
  Patients with a creatinine clearance of 30-50 mL/min must commence treatment with CAPE at 75% of the full dose. Dose modifications for hematologic, skin or GI toxicity will be based on the worst toxicity observed during the previous cycle. After recovery, standard dose adjustments for capecitabine toxicity should be applied. The dose of capecitabine should be reduced by 25% in subsequent cycles for the following toxicities: febrile neutropenia, grade 4 thrombocytopenia, or failure of hematological recovery to neutrophils ≥1500 /mL and platelets ≥75000/mL within 2 weeks of the scheduled start of the next treatment cycle; grade 3-4 mucositis, hand-foot syndrome, diarrhea, or nausea or vomiting in spite of optimal antiemetic prophylaxis.
3. Oxaliplatin;
  If a second dose reduction of 5FU is performed, oxaliplatin dose must also be reduced by 25%. Oxaliplatin neurotoxicity should be assessed before every oxaliplatin dose. If neurotoxicity is grade 3, oxaliplatin dose should be reduced by 25%. If toxicity is grade 4, oxaliplatin should be discontinued permanently. For oxaliplatin infusion reactions grade 2 or less occur, oxaliplatin can have its infusion time extended until 6 hours and the patient must receive pre medications such as H1 antagonists, H2 antagonists and corticosteroids. If it recurs or is graded 3 or more, oxaliplatin administration must be suspended. Where it is available, desensitization protocols can be applied, 5FU may continue even if oxaliplatin is discontinued.

### RT Toxicity and Stopping Rules.

Toxicity will be assessed and recorded according to the CTCAE v4.0 acute radiation morbidity scoring criteria. (Table 1).

**Table 1:** Stopping rules for radiotherapy during chemoradiation

Adverse event	Definition	Action
Diarrhea	Grade 4	should be interrupted until the treatment-related symptoms have been reduced and parenteral support is no longer necessary
Other gastro- Intestinal toxicity	Grade 4	should be interrupted and restarted according to the patients' condition

CTC v 4.0 https://ctep.cancer.gov/protocoldevelopment/electronic_applications/ctc.htm#ctc_40

## Assessment of response

Assessment of tumor response will be performed at 12 weeks (and 18 weeks from last date of radiation therapy if cCR or near-complete response is detected at 12 weeks). All patients will undergo endoscopic reassessment, DRE and high-resolution MR. Endoscopic biopsies will be at the discretion of the attending surgeon/endoscopist.

Patients with complete or near-complete clinical response at 12 weeks will be recommended reassessment at 18 weeks from RT. Patients with clinically overt incomplete clinical response at 12 or 18 weeks will be referred to immediate radical surgery.

## Randomization

Individuals will be randomized and allocated at a 1:1 ratio to the two groups (single-drug and combination with oxaliplatin) using a permuted block design with a random block size of 4, 6, and 8. (1–3).

A randomization list will be generated electronically using appropriate software immediately after being considered eligible.

## Protocol blinding

Patients and attending phisicians will not be blinded to the treatment arm randomized for each patient. However, as a strategy to reduce investigator’s expectations and reduce inherent bias, the central committee will be blinded to the treatment arm. It is expected that blinding the central committee, who will be responsible for assessing the primary endpoint, any bias related to the investigator’s expectation about the treatment arm and the chances of Watch and Wait will be nulled.

## Sample size calculation

The primary endpoint (decision to WW due to cCR/near-complete response) was observed in 55% and 85% at 12 weeks (in contrast to the 18 weeks used in the present trial) among patients with early cT3 and cT2 rectal cancer respectively [29]. In this study, there was a distribution of 66% of cT3 and 33% cT2. Therefore, response rates appear to be highly dependent on exact T-stage distribution. Considering the expected inclusion of more advanced disease (late mrT3) we expect 40% cCR/near-complete response in the control arm. A similar difference 60% versus 40% was achieved in the preliminary report of the outcomes of the OPRA trial. This difference in TME-free survival at 3 years was statistically significant favoring patients undergoing nCRT with cCT in comparison to nCRT preceeded by induction chemotherapy (both regimens incorporating oxaliplatin). In this setting, if experimental results in ≥ 60% cCR/near-CR, the study will be considered POSITIVE. The incorporation of oxaliplatin to a consolidation CRT regimen that results in ≥ 20% increase in cCR/near-CR exceeds the potential disadvantages of treatment-related toxicity.

We will assume that the primary outcome will occur in 40% of individuals in control group and 60% in the 2-drug consolidation chemotherapy group, which corresponds to an absolute difference in proportions of 20%. It is estimated that a sample of 194 (97 per group) provides 80% statistical power to detect this difference at a significance level of 5% using the Chi-square test and assuming a two-sided significance hypothesis and considering a 1:1 allocation. The estimated dropout rate is 10% in each group, so we would have 216 individuals (108 per group). The sample size calculation was performed using SAS 9.4 (PROC POWER procedure).

## Time-table

Patient accrual: 2 years and 6 months.

Patient/institution/year: 5–6 (20 institutions: 100–120/year – 2 years n = 200–240).

## Interim analysis

If the arm of combination with oxaliplatin (2-drug chemotherapy) during consolidation shows ≥ 25% response rate difference compared to single-drug arm after 72 patients, study will be interrupted (efficacy). If the arm of 2-drug consolidation chemotherapy shows less than 5% response rate difference compared to single-drug arm after 72 patients, the study will be interrupted.

## Discussion

Complete primary tumor regression has become a relevant endpoint in rectal cancer management. Achievement of a complete clinical response to neoadjuvant treatment strategies has provided the opportunity to avoid major abdominal surgery, its associated morbidity and the requirement for temporary or definitive stomas. Long-term data suggests that nearly 70% of patients who achieve a cCR will never require radical surgery [5, 8, 9]. In addition, in 30% of these patients that develop local regrowth salvage resection is successful in the vast majority of patients leading to excellent local disease control and survival [7, 30].

In this setting, changes in neoadjuvant treatment regimens may now be driven by the attempt to increase response rates. Most studies at this point, presented nearly 25% cCR rates among centers practicing WW and using standard CRT regimens similar to the experimental arm of the German trial (2 cycles of 5FU-based chemotherapy) [9]. Within this context, several treatment alternatives have been investigated in order to improve response rates to allow for organ-preservation. Initial retrospective studies (before introduction of total neoadjuvant therapy concept—TNT) suggested that the inclusion of additional chemotherapy agents (in addition to 5FU/fluoropyrimidines and concomitant to RT) to nCRT regimens would significantly increase pCR rates [17]. Oxaliplatin was the most common additional chemotherapy agent added to 5FU. Unfortunately, subsequent randomized clinical trials failed to demonstrate significant increases in pCR rates when oxaliplatin was added to standard nCRT regimens. Instead, a significant increase in treatment related toxicity was observed [22].

A single phase 2 study performed using additional cycles of bolus 5FU aimed at improving cCR (instead of pCR) rates [16, 21, 29]. This study incorporated 4 additional cycles of bolus 5FU infusion (to the usual 2 cycles) to be delivered during RT but also during the “resting” period. Surprisingly, cCR rates were nearly 50% of all patients treated including tumors with baseline T2/T3 rectal cancer. While the increase in cCR rates could have been attributed to the increase in number of cycles of chemotherapy delivered during and after RT completion (“consolidation” chemotherapy even though not named as such at the time), one additional change in the regimen could have also contributed to the increase in cCR rates: RT dose escalation was also incorporated to this nCRT regimen (50.4 Gy to 54 Gy). Therefore, it became impossible to establish a direct cause-effect relationship between cCR rates and consolidation chemotherapy or RT dose-escalation.

Another prospective non-randomized study also suggested the potential effects of consolidation chemotherapy to response in rectal cancer. The “timing” trial included patients with locally advanced disease into 4 different arms (sequentially, not randomized) [20, 31, 32]. The primary objective of the study was to investigate progressive longer interval periods between RT completion and surgery in response: 6, 12, 18 and 24 weeks. However, patients included in the 12, 18 and 24 received consolidation chemotherapy with mFOLFOX (2,4 and 6 cycles respectively) resulting in significant increases in pCR rates (pCR 25%, 30% and 38% respectively) compared to no consolidation chemotherapy (pCR 18%). Again, while the increase in pCR rates could have been attributed to the increase number of chemotherapy cycles (consolidation), the effect of prolonged intervals in time could also have contributed to this observation.

Finally, with the introduction of the TNT concept – providing adjuvant chemotherapy immediately before nCRT (induction) or after nCRT (consolidation), initial experiences (using FOLFOX as consolidation) suggested an increase in complete response and in the chances of organ preservation among these patients [33]. One prospective randomized study (OPRA) presented the preliminary outcomes of the comparison between induction and consolidation chemotherapy (FOLFOX). While there was no difference in 3-yr disease free survival rates between arms, organ-preservation rates was significantly better for consolidation chemotherapy when compared to induction chemotherapy.

In summary, there is evidence to support that consolidation chemotherapy may contribute to improve response rates in rectal cancer following nCRT. Both 5FU-only and 5FU/Oxaliplatin-based chemotherapy consolidation regimens have shown promising results [20, 21]. However, no study addressed the benefit of oxaliplatin during consolidation to improve response rates. While previous studies incorporating oxaliplatin were negative and associated with increased toxicity, all of these studies used oxaliplatin in concomitance to RT.

For these reasons, we decided to design a study to address the impact of 2 different consolidation chemotherapy regimens in complete primary tumor response to treatment in rectal cancer [34]. The findings of the present study may allow for a definitive recommendation of specific consolidation regimens, particularly when the primary purpose of the use of neoadjuvant therapy is to achieve a complete clinical response and offer organ-preservation to these patients.

The decision to exclude patients with mrT4 disease was due to the fact that these patients (T4 disease irrespective of N stage) are at high-risk for development of subsequent metastases. Currently, guidelines recommend the use of combined chemotherapy regimens (5FU and oxaliplatin-based) in the adjuvant setting for such patients with colorectal cancer. (Baxter J Clin Oncol 2021 ASCO guideline) [35] Therefore, patients with mrT4 allocated in the 5FU alone-based study arm were at significant risk for undertreatment. For these reasons, the presence of mrT4 disease is considered an exclusion criterion and patients are routinely recommended TNT regimens with combination (5FU and oxaliplatin-based) chemotherapy regimens across all participating centers.

Finally, several reasons support our decision to use long-course instead of short-course RT in both treatment arms. First, while preliminary data suggests short-course RT may also lead to significant chances for a pCR (particularly in the setting of consolidation chemotherapy), less is known about the chances of a cCR (primary endpoint of our study) [36]. Since most of the studies with short-course RT and cCR have also included patients treated with long-course, little is known about the denominators in cCR rates and therefore complicating estimates of sample sizes required for a prospective study [37]. Second, there is robust long-term data for organ-preservation after a cCR for long-course RT while in short-course RT such data is yet unavailable [38]. Finally, considering the recently published concerning local failure rates associated with short-course RT arm in the RAPIDO study, the consortium believes that until more mature data is available (including from ongoing studies), long-course is still the preferred option for patients where cCR is a relevant clinical endpoint [39].

## Data Availability

Not applicable.

## Abbreviations

APR: Abdominal perineal resections
cCR: Clinical complete response
cCT: Consolidation chemotherapy
CT: Computed tomography
CTV: Clinical target volume
DRE: Digital rectal examination
EMVI: Extramural vascular invasion
GI: Gastrointestinal
GTV: Gross target volume
ISR: Intersphincteric resections
MR: Magnetic resonance
MRF: Mesorectal fascia
nCRT: Neoadjuvant chemoradiation
pCR: Pathological complete response
PTV: Planning target volume
RT: Radiotherapy
TME: Total mesorectal excision
TNT: Total neoadjuvant therapy
TRG: Tumor regresion grade
WW: Watch and Wait
